# Lactylation in tumor: mechanisms and therapeutic potentials

**DOI:** 10.3389/fimmu.2025.1609596

**Published:** 2025-06-16

**Authors:** Dandan Wang, Hao Rong, Ke Ma, Jun Peng

**Affiliations:** Department of Thoracic Surgery, Sichuan Clinical Research Center for Cancer, Sichuan Cancer Hospital & Institute, Sichuan Cancer Center, University of Electronic Science and Technology of China, Chengdu, Sichuan, China

**Keywords:** lactate, lactylation, post-translational modifications, tumor metabolism, tumor microenvironment

## Abstract

Lactate, a central product of glucose metabolism, plays a vital role in energy supply and signal transduction, and it also participates in gene transcription regulation through lactylation. Metabolic reprogramming is a key feature of tumor cells and highlights the important role of lactylation in cancer development. Recent studies have emphasized the significant regulatory roles of lactylation in cancer, suggesting that it may serve as a potential target for treatment. This review discusses the mechanisms, regulation, and functions of lactylation in cancer. It also explores the possible significance of lactylation as a marker for the diagnosis and therapy of tumor, and evaluates the therapeutic prospects of targeting lactylation. While the precise mechanisms of lactylation in cancer regulation require further investigation, its significant influence indicates promising avenues for future research.

## 1 Introduction

In the biological metabolic network, the flux of pyruvate, produced from glucose through enzymatic reactions, is tightly regulated by oxygen levels. Under aerobic conditions, pyruvate is converted into acetyl-CoA by the enzyme pyruvate dehydrogenase (PDH). This molecule then enters the tricarboxylic acid cycle, facilitating efficient adenosine triphosphate (ATP) production through oxidative phosphorylation. In low-oxygen environments, pyruvate is converted by lactate dehydrogenase (LDH) into lactate (1). Lactate has traditionally been considered a byproduct of anaerobic glycolysis. However, recent research has gradually uncovered the various biological roles of lactate: it can serve as an energy reserve and a key precursor for gluconeogenesis, and also participate in cellular signaling transduction by modulating redox balance (2–4). Notably, as a signaling molecule for the redox state between cells and tissues, it possesses biological attributes beyond those of a metabolic intermediate (2).

In 1956, Warburg identified a process called aerobic glycolysis, or the Warburg effect. This process shows that cancer cells mainly convert glucose through glycolysis instead of oxidative phosphorylation, even when oxygen is present, resulting in a significant accumulation of lactate (5). Tumor-produced lactate is transported across the cell membrane into the extracellular space via monocarboxylate transporters (MCTs), which acidify the tumor microenvironment (TME). Research has confirmed that the acidic microenvironment can induce apoptosis of natural killer (NK) cells and natural killer T (NKT) cells. It also activates the reprogramming of tumor-associated macrophages (TAM), leading to immune suppression and the development of metastasis (6–9).

In 2019, the identification of lysine lactylation (Kla) as a novel type of post-translational modification (PTM) marked a paradigm shift in research regarding lactate functions (10). Lactylation dynamically regulates the biological activity of target proteins by covalently attaching lactyl groups to lysine residues. At the chromatin level, histone lactylation can promote chromatin relaxation by altering the surface charge of nucleosomes, thereby regulating the transcriptional activity of specific genes. At the non-histone level, lactylation modification can regulate protein stability and function. This review offers a thorough overview of the molecular processes involved in lactylation and its role in tumor development. It also explores the potential of targeting lactylation for therapeutic use.

## 2 Molecular basis and regulation of lactylation

### 2.1 Biochemical mechanisms

**Figure 1:**
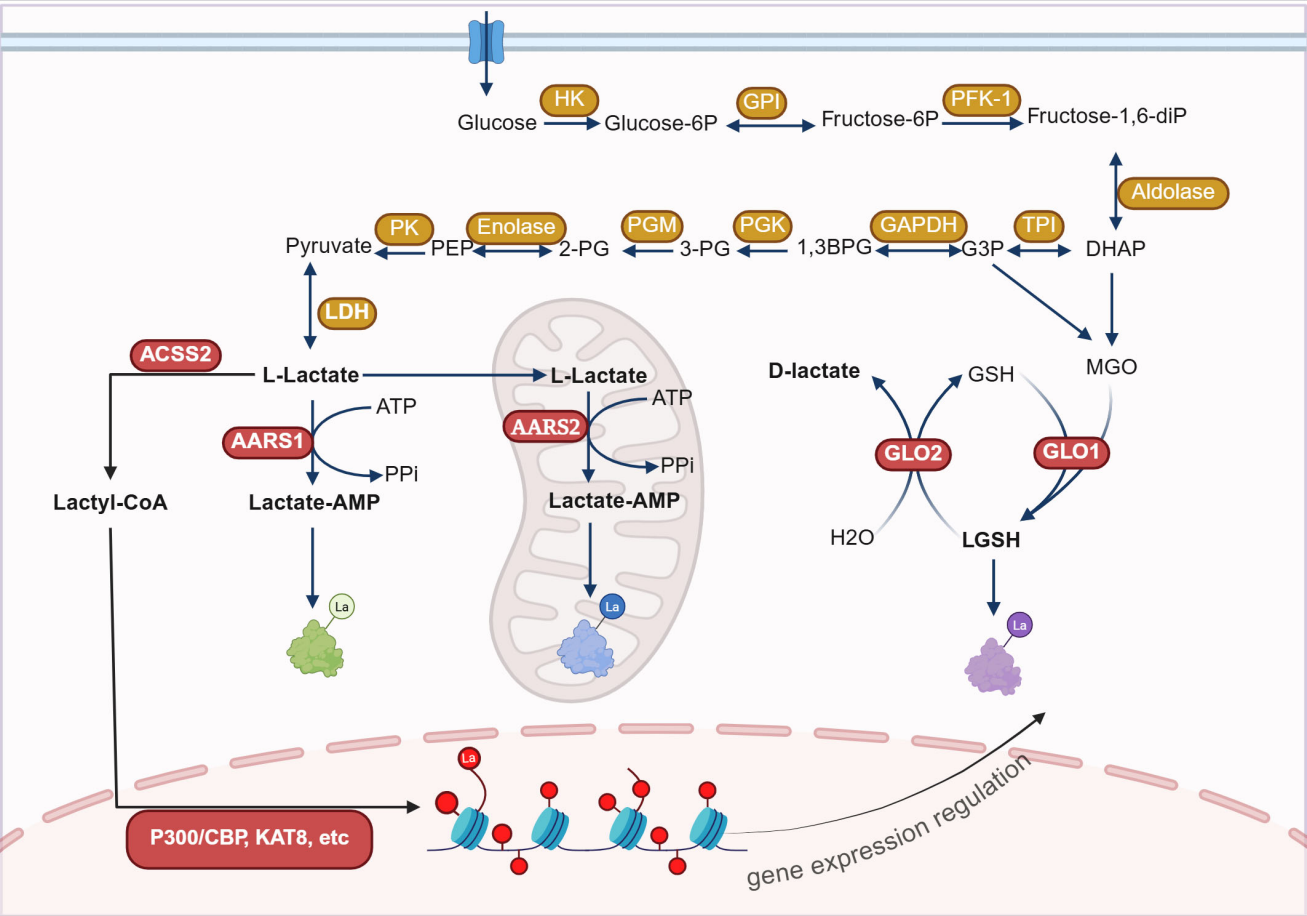
The mechanisms of lactylation modification. Lactylation modification occurs through enzymatic reactions using lactyl-CoA as the substrate and non-enzymatic reactions using LGSH as the substrate. The enzymatic lactylation process is mediated by enzymes such as P300, AARS1, and AARS2, and can take place in the nucleus, cytoplasm, and mitochondrial matrix. Non-enzymatic reactions can occur in the cytoplasm.HK, Hexokinase; GPI, Glucose-6-phosphate isomerase; PFK-1, Phosphofructokinase-1; TPI, Triose phosphate isomerase; GAPDH, Glyceraldehyde-3-phosphate dehydrogenase; PGK, Phosphoglycerate kinase; PGM, Phosphoglycerate mutase; PK, Pyruvate kinase; LDH, Lactate dehydrogenase; ACSS2, Acetyl-CoA synthetase 2; AARS1, Aminoacyl-tRNA synthetase 1; GLO1, Glyoxalase 1; ATP, Adenosine triphosphate; GSH, Glutathione; MGO, Methylglyoxal; P300, E1A binding protein p300; CBP, CREB-binding protein; KAT8, lysine acetyltransferase 8. Figure created in https://BioRender.com.

Lactylation is an emerging type of protein PTM, and its mechanisms are categorized into enzymatic and non-enzymatic pathways. L-lactate and D-lactate, as different chiral forms of lactate, participate in the enzymatic lactylation process and the non-enzymatic process, respectively (**Figure 1**).

Enzymatic lactylation mainly occurs through two pathways: the L-lactyl-CoA pathway and the lactate-AMP pathway. In the L-lactyl-CoA pathway, L-lactate generated during glycolysis is converted into L-lactyl-CoA by lactyl-CoA synthetase (such as acetyl-CoA synthetase 2 (ACSS2)). Then the lactyl group is transferred to the lysine residues of target proteins, catalyzed by modification enzymes (such as acetyltransferases), resulting in lactylation modification (Kla) (10, 11). In the lactyl-AMP pathway, lactate binds with aminoacyl‐tRNA synthetase 1 (AARS1) or AARS2, forming a lactate-AMP intermediate in an ATP-dependent manner, which subsequently modifies target proteins by adding the lactyl group to lysine sites (12–14). Enzymatic lactylation primarily targets histones, regulating gene transcription by altering chromatin spatial conformation (10, 15). It can also modify non-histone proteins, influencing cellular metabolism and signal transduction (16–21).

D-lactate is involved in the non-enzymatic lactylation modification pathway. The glycolytic byproduct methylglyoxal (MGO) conjugates to glutathione (GSH) under the action of glyoxalase 1 (GLO1), generating lactylglutathione (LGSH). LGSH is then catalytically hydrolyzed by GLO2, cycling glutathione and generating D-lactate. LGSH acts as a precursor for lactylation, leading to K(D-la) modification of proteins. Unlike K(L-la), K(D-la) modification primarily affects non-histone proteins, especially glycolytic enzymes, thereby inhibiting glycolysis by reducing their activity (22). A recent study revealed that immune activation significantly reduces GLO2 expression in immune cells, such as macrophages and dendritic cells. This decrease causes an accumulation of the substrate S-D-lactylglutathione (SLG) within the cytoplasm, which induces K(D-la) modification of proteins that are dependent on adjacent cysteine residues. Specifically, D-lactylation modification at the K310 site of the RelA can significantly inhibit the transcriptional activity of NF-κB, thereby attenuating inflammatory responses and maintaining immune homeostasis (23).

### 2.2 Metabolic and transport regulation

The dynamic balance of lactylation modifications is co-regulated by lactate metabolism, transport systems, and lactyltransferase/delactylases systems.

Lactate serves as the direct substrate for lactylation, with its concentration positively correlating with the degree of lactylation (10). The buildup of lactate is affected by the metabolic condition, hypoxic environment, and lactate transport capacity (14, 24).

#### 2.2.1 Production of lactate and lactylation modification

Lactate is mainly produced via the glycolysis pathway, and the activity of its key enzyme LDH directly affects the level of lactylation modification. Research indicates that lactate dehydrogenase A (LDHA) significantly enhances the lactylation modification level at the H3K18 site in the JunB proto-oncogene (*JunB*) promoter region in osteoblasts by promoting lactate production, thereby activating *JunB* transcription and inducing osteoblast differentiation. Further experiments confirmed that knocking down LDHA significantly decreased histone lactylation at the *JunB* promoter, but adding external lactate reversed this reduction (25). In a hypoxic pulmonary hypertension model, inhibiting LDH activity significantly reduced the level of histone lactylation (26). Using sodium dichloroacetate and oxalate to inhibit the activity of PDH and LDH, thereby blocking lactate production, can significantly reduce histone lactylation levels. In contrast, using the inhibitor rotenone to inhibit the mitochondrial respiratory chain enhances glycolysis and increases lactylation modification, suggesting a direct link between lactate production and lactylation modification (10). Furthermore, in early models of myocardial infarction, the metabolic pattern of monocytes transitions from oxidative phosphorylation to glycolysis dependence, resulting in lactate accumulation and a marked increase in cellular lactylation levels. Research has found that lactylation modification at the H3K18 site promotes post-infarction cardiac repair and improves cardiac function by activating the transcriptional expression of repair genes such as leucine-rich alpha-2-glycoprotein 1 (*LRG1*) and *VEGF-α (* 27). These research findings consistently indicate that the activity of the glycolysis pathway directly drives the increase in lactylation modification levels.

#### 2.2.2 The transport of lactate and lactylation modification

Lactate is primarily transported across membranes by MCTs, particularly MCT1 and MCT4 (28). MCT1 is widely expressed in cardiac tissue, skeletal muscle, and liver tissue. It primarily facilitates the uptake of lactate (28, 29). MCT4 is expressed in cells under hypoxic conditions and those with high glycolytic activity, and it is primarily responsible for lactate efflux (28, 30). In the TME, glycolytic cancer cells located far from blood vessels release lactate through MCT4. In contrast, oxidative cancer cells near blood vessels take up lactate through MCT1 for oxidative phosphorylation, creating a lactate shuttle mechanism (31). Research shows that lactate increases the level of lactylation in cortical neurons in a dose-dependent manner. Additionally, the selective MCT1/2 inhibitor, AR-C155858, can block this process (17). Furthermore, in a sepsis model, macrophages uptake lactate through MCT, driving lactylation modification on high mobility group box 1 (HMGB1), thereby promoting disease progression. The MCT inhibitor α-cyano-4-hydroxycinnamic acid (CHC) can effectively block this process (20). These studies highlight the essential function of lactate transport in regulating lactylation.

### 2.3 Enzymatic modifiers

#### 2.3.1 Regulation of lactylation by modification enzymes

The regulation of lactylation modification depends on the dynamic balance between lactyltransferase and delactylases (**Table 1**). P300 was the first lactylation modification enzyme to be discovered. P300 and its homolog CBP can catalyze lactylation modification of the promoter regions of *HMGB1* and YTH N6-methyladenosine RNA binding protein 2 (*YTHDF2*), thereby enhancing the activation of the relevant genes (20, 32). In colorectal cancer, lysine acetyltransferase 8 (KAT8) induces lactylation at the K408 site of the eukaryotic translation elongation factor 1 alpha 2 (eEF1A2). This modification enhances protein translation efficiency and promotes tumor progression (33). Similarly, KAT7 has been identified as an enzyme involved in lactylation modification (34). Furthermore, histone acetyltransferase binding to ORC1 (HBO1/KAT7) preferentially catalyze the lactylation of H3K9 (34). In a myocardial infarction model, the silencing of general control of amino-acid synthesis 5 (GCN5) significantly inhibits H3K18la modification and its target gene expression, suggesting that it may function as an enzyme involved in lactylation modification (27). In *Escherichia coli*, YiaC is considered a lactylation modification enzyme that facilitates the movement of the lactyl group from lactyl-CoA (35). Notably, AARS1 and AARS2 directly mediate lactylation modification via the lactate-AMP pathway. AARS1 is predominantly cytoplasmic, while AARS2 is highly enriched in the mitochondria (12–14). Given the extremely low concentration of Lactyl-CoA in tumor cells—approximately 1/1000th compared to Acetyl-CoA—AARS1 and AARS2 may serve as the primary enzymes mediating lactylation in these cells (36).

**Table 1:** The modification enzymes of lactylation.

Lactyltransferase	Substrate	Subcellular location	Species	Reference
P300/CBP	Lactyl-CoA	Nucleus	–	(10, 20, 32)
KAT8	Lactyl-CoA	Nucleus	–	(33)
HBO1/KAT7	Lactyl-CoA	Nucleus		(34)
GCN5	Lactyl-CoA	Nucleus	–	(27)
YiaC	Lactyl-CoA	Nucleus	*Escherichia coli*	(35)
AARS1	Lactate‐AMP	Cytoplasm	–	(12, 13)
AARS2	Lactate‐AMP	Mitochondrion	–	(14)

P300, E1A binding protein p300; CBP, CREB-binding protein; KAT8, Lysine acetyltransferase 8; HBO1/KAT7, Histone acetyltransferase binding to ORC1; GCN5, General control of amino-acid synthesis 5; YiaC, YiaC protein; AARS1, Aminoacyl-tRNA Synthetase 1.

#### 2.3.2 Regulation of lactylation by demodification enzymes

The dynamic reversibility of lactylation modification depends on the activity of demodification enzymes. Lactylation demodification enzymes mainly include histone deacetylases (HDAC) 1–3 and sirtuin (SIRT) 1-3 (**Table 2**). *In vitro* screening and functional experiments have shown that HDAC1 and HDAC3 are the primary enzymes responsible for delactylating lysine residues on histones in cells (15). In the study of *Toxoplasma gondii*, the application of antibodies targeting TgHDAC2, TgHDAC3, and TgHDAC4 was found to elevate the total amount of protein lactylation, suggesting that TgHDAC2, TgHDAC3, and TgHDAC4 also exhibit enzymatic activity for lactylation demodification (37). At the non-histone level, based on the genetic coding of lactylated lysine, particularly e-N-L-lactyllysine (LacK) in both bacterial and mammalian cells, SIRT1 and SIRT3 have been identified as potential demodification enzymes, reversing lactylation modification by removing the lactyl group (38). Additionally, SIRT2 is recognized as a lactylation demodifying enzyme. Its knockdown increases H4K8la levels and induces the transcription of the serpin family G member 1 (*SERPING1*) and transient receptor potential cation channel, subfamily V, member 4 (*TRPV4*) genes, which promotes the proliferation and migration of neuroblastoma cells (39). In *Escherichia coli*, the SIRT family protein CobB, which depends on nicotinamide adenine dinucleotide (NAD), regulates the de-lactylation of proteins (35). These studies reveal the crucial role of lactylation demodification enzymes in the dynamic balance of lactylation.

**Table 2:** The potential demodification enzymes of lactylation.

Delactylases	Subcellular location	Species	Evidence	Reference
HDAC1	Nucleus	–	Overexpression of it reduces H4K5la levels; knockdown of it increases H4K5la levels	(15)
HDAC2	Nucleus and cytoplasm	–	Overexpression of it reduces H4K5la levels; its antibodies can increase total protein lactylation in *Toxoplasma gondii*	(15, 37)
HDAC3	Nucleus and cytoplasm	–	Overexpression of it reduces H4K5la levels; inhibition of its expression increases H4K5la levels in HeLa cells and total protein lactylation in *Toxoplasma gondii*	(15, 37)
HDAC4	Cytoplasm	*Toxoplasma gondii*	Its antibodies can increase total protein lactylation	(37)
SIRT1	Nucleus and cytoplasm	–	Probes based on the genetic encoding of lactylated lysine reveal it as a potential demodification enzyme for non-histone proteins	(38)
SIRT2	Nucleus and cytoplasm	–	Knockdown of it increases H4K8la levels	(39)
SIRT3	Mitochondrion	–	Probes based on the genetic encoding of lactylated lysine reveal it as a potential demodification enzyme for non-histone proteins	(38)
CobB	Cytoplasm	*Escherichia coli*	Strains overexpressing CobB exhibit significantly reduced Kla levels	(35)

HDAC, Histone deacetylases; SIRT, Sirtuin; CobB, CobB protein.

In conclusion, the modification enzymes for lactylation are not fully identified, and their recognition and functional mechanisms need further investigation. The enzymatic network of lactylation and their functional studies in tumors may offer a theoretical foundation for developing related therapeutic approaches.

## 3 Identification of lactylation sites

In 2019, Zhang et al. used mass spectrometry to discover 26 histone lactylation sites in human MCF-7 cells and 18 in mouse bone marrow-derived macrophages (BMDM) (10). Subsequently, other researchers have also identified histone lactylation sites (**Table 3**) (6, 10, 16, 18, 19, 37, 40–47). In addition, researchers have gradually identified non-histone lysine lactylation sites (**Table 4**) (16, 19, 35, 37, 46, 48–52).

**Table 3:** Lactylated protein sites of histones in different species.

Species	Histone	Site	Reference
Human	H2A	K11,K13,K115	(10, 40–43)
	H2A.Z	K32,K36,K44,K165	
	H2B	K5,K11,K15,K16,K20,K23,K43,K85,K108,K116,K120	
	H3	K9,K14,K18,K24,K33,K56,K62,K79,K123	
	H4	K5,K8,K12,K16,K31,K77,K91	
Mouse	H2A	K4,K9,K11,K115	(6, 10, 42, 44, 45)
	H2B	K5,K11,K15,K16,K20,K85,K108	
	H3	K14,K18,K23,K27,K56	
	H4	K8,K12,K31,K91	
*Toxoplasma gondii*	H2A	K5,K137,K142	(37, 46)
	H2A.Z	K5,K9,K17,K23,K142,K150	
	H2A.1	K73	
	H2A.X	K127	
	H2B	K37,K47,K70,K77,K99	
	H2B.Z	K3,K8,K18,K104	
	H2B.b	K46,K98	
	H3	K14,K23,K27,K56,K122	
	H4	K12,K31	
*Trypanosoma brucei*	H2A	K5,K21,K116	(18)
	H2A.Z	K32,K36,K44,K165	
	H2B	K5,K97	
	H2B.v	K8,K20,K28	
	H3	K24,K33,K62	
	H4	K78	
*Botrytis cinerea*	H2A	K5,K23	(16)
	H2B	K15,K48,K122	
	H3	K123	
Rice	H2B	K41,K60,K66,K114,K136,K144	(19)
	H3	K9,K14,K18,K56	
	H4	K5,K8,K16#x3001;K31	
Sheep	H3	K18	(47)

**Table 4:** Lactylated protein sites of non-histones in different species or tissues.

Species/tissues	Lactylated sites	Biological pathways	Reference
*Toxoplasma gondii*	1964 Kla sites in 955 proteins	mRNA splicing, glycolysis, aminoacyl-tRNA biosynthesis, and RNA transport	(37)
*Toxoplasma gondii*	983 Kla sites in 523 proteins	protein translation process	(46)
*Botrytis cinerea*	273 Kla sites in 166 proteins	fungal virulence, host adherence, signal transduction, primary nutrients transduction, molecular chaperons function, and ribosomal translation	(16)
*Escherichia col*	478 Kla sites in 1047 proteins	bacterial metabolism and biosynthesis	(35)
Rice	638 Kla sites in 342 proteins	central carbon metabolism, protein biosynthesis, grain development, and nutrient accumulation	(19)
Verrucosispora strains	636 Kla sites in 420 proteins	protein synthesis process and fungal pathogenicity	(48)
Human liver cancer tissues	9256 Kla sites	tricarboxylic acid cycle, carbohydrate metabolism, amino acid metabolism, fatty acid metabolism, and nucleotide metabolism	(49)
Cerebral ischemia-reperfusion injury rats	1003 Kla sites in 469 proteins	Ca^2+^ signaling pathway, cGMP-PKG signaling pathway, human T-cell leukemia virus1 infection, cellular senescence, MAPK signaling pathway, human cytomegalovirus infection, oxytocin signaling pathway, and vascular smooth muscle contraction	(50)
Human gastric cancer tissues	2375 Kla sites in 1014 proteins	RNA splicing	(51)
Human lung tissues	724 Kla sites in 451 proteins	–	(52)

Traditionally, Kla has been detected by mass spectrometry-based methods. Proteins are extracted from biological samples and then digested with enzymes. Lactylated peptides are enriched using pan-lactylation antibodies or lactylation site-specific antibodies, followed by liquid chromatography–mass spectrometry analysis of lactylation sites. The lactylation sites are further confirmed by biochemical and chemical methods, such as peptide synthesis and isotopic labeling (16). Due to the cumbersome procedure, time-consuming process, and high requirements for sample purity and quality of mass spectrometry, researchers have gradually developed other methods for detecting Kla sites. By combining few-shot learning with a hybrid system, researchers designed a lactylation site predictor called “FSL-Kla”. This predictor computes Kla sites from protein sequences, enabling the identification of potential lactylation modification sites on proteins (53). It is capable of learning and predicting Kla sites based on limited sample data. However, its heavy reliance on manually designed hypothesis spaces and search strategies somewhat restricts its universality and flexibility. The bioorthogonal chemical probe YnLac has been used to identify protein lactylation in mammalian cells, uncovering four previously unknown lactylation sites in non-histone proteins, such as high mobility group nucleosome binding domain 1 (HMGN1), nucleophosmin 1 (NPM1), programmed cell death protein 4 (PDCD4), and polyhomeotic-like domain protein 3 (PHF3) (44). It can detect Kla sites in living cells and monitor the dynamic changes of lactylation in real time with high specificity. However, the synthesis and operation processes are complex, and it is currently limited to detecting Kla sites in mammals. In tandem mass spectrometry analysis, cyclic iminium ions have demonstrated strong sensitivity and specificity in detecting protein lactylation modifications. Based on this, researchers have uncovered numerous novel lactylated proteins and their corresponding sites from human proteome data. Notably, lactylation modifications are abundant on enzymes involved in the glycolytic pathway. For instance, the lactylation of the K147 site on fructose-bisphosphate Aldolase A (ALDOA) reduces its activity (54). This discovery unveils a negative feedback mechanism in the glycolytic pathway: when glycolysis is overly activated, leading to lactate accumulation, the activity of upstream enzymes like ALDOA is suppressed through lactylation, thereby decreasing glycolytic flux and lactate levels. This method is primarily applied to the study of the human proteome. Detection of Kla sites in other species may require further validation and optimization. Recently, a human Kla site predictor (PBertKla) based on the protein large language model has been proposed. On an independent validation dataset, PBertKla demonstrated excellent prediction performance with an accuracy of 80.3%, sensitivity of 78.7% and specificity of 82.0%. In addition, its matthews correlation coefficient (MCC) was 0.607, the area under the receiver operating characteristic curve (AUC) was 0.884, and the area under the precision rate recall curve (AUPRC) was 0.866. These metrics demonstrate the tool’s strong predictive capabilities (55). However, the potential of PBertKla to identify Kla sites in other species has not been fully explored.

As lactylation research progresses, the number of identified lactylation sites continues to grow. Analyses of biological lactylation sites and enriched pathways for lactylated proteins indicate that lactylation modifications are widespread in organisms and may significantly impact protein function and biological metabolism.

## 4 Molecular mechanisms of lactylation modification in the pathological progression of tumors

Metabolic reprogramming functions as a key process for cellular adjustment to environmental changes. As a key metabolic intermediate, lactate participates in the malignant progression of tumors through the novel PTM mechanism of lactylation.

### 4.1 Regulatory network of lactylation in digestive system tumors

**Figure 2:**
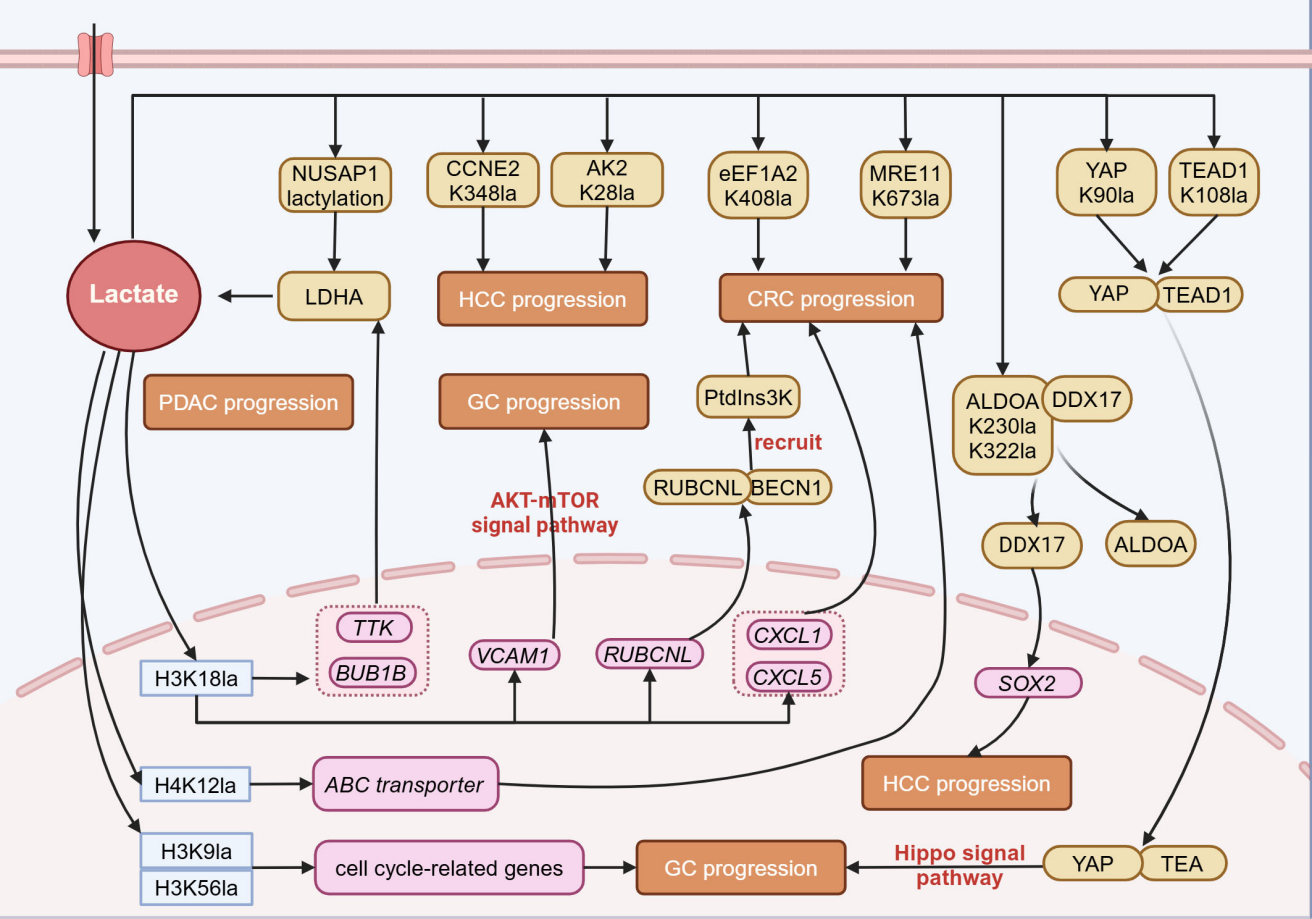
Lactylation in digestive system tumors. In digestive system tumors, lactate can participate in tumor progression through lactylation modification. Lactylation at histone sites such as H3K18, H4K12, H3K9, and H3K56 can promote tumor progression by regulating gene expression. Lactylation of non-histone proteins also participates in tumor progression by altering protein structure and interactions with other proteins. NUSAP1, Nucleolar and spindle associated protein 1; CCNE2, Cyclin E2; AK2, Adenylate Kinase 2; eEF1A2, Eukaryotic translation elongation factor 1 alpha 2; MRE11, Meiotic recombination 11 homolog A; YAP, Yes-associated protein; TEAD1, TEA domain transcription factor 1; LDHA, Lactate dehydrogenase A; ALDOA, Aldolase A; DDX17, DEAD-box helicase 17; Ptdins3K, Phosphatidylinositol-3-Kinase; RUBCNL, Rubicon like autophagy enhancer; BECN1, Beclin 1; TTK, TTK protein kinase; BUB1B, BUB1 mitotic checkpoint serine/threonine kinase B; VCAM1, Vascular cell adhesion molecule 1; CXCL1, Chemokine (C-X-C Motif) Ligand 1; SOX2, SRY-Box Transcription Factor 2; PDAC, Pancreatic ductal adenocarcinoma; HCC, Hepatocellular carcinoma; GC, Gastric cancer; CRC, Colorectal cancer. Figure created in https://BioRender.com.

Emerging evidence indicates that lactylation modification is closely associated with the initiation and progression of gastrointestinal tumors, including gastric cancer, hepatocellular carcinoma, pancreatic ductal adenocarcinoma, colorectal cancer, esophageal cancer, and cholangiocarcinoma (**Figure 2**).

#### 4.1.1 Gastric cancer

Gastric cancer (GC) originates from the epithelial cells that line the stomach’s mucosal layer. It has one of the highest incidence and mortality rates among all cancer types (56). Several factors contribute to the development of GC, including smoking, alcohol use, and infection with Helicobacter pylori (57). Recent studies have revealed the crucial role of metabolic reprogramming in the pathophysiology of GC. Research has found that compared to adjacent normal tissues, GC tissues show a significant increase in glycolytic metabolites, such as lactate and pyruvate. Glycolysis inhibitors have been shown to notably reduce the proliferation of GC cells and improve their sensitivity to chemotherapy (58, 59). β-Arrestin 1 (ARRB1) can induce metabolic reprogramming in GC, shifting from mitochondrial OXPHOS to glycolysis, thereby promoting the proliferation of gastric cancer cells (60).

Lactylation has recently been demonstrated to promote the progression of GC. Through the analysis of bioinformatic data from GC, a significant correlation has been identified between high lactylation-related gene scores and low overall survival (OS) rates, high tumor grades, and lymph node metastasis. Individuals with elevated lactylation scores show more prominent immune cell infiltration, increased genetic instability, higher risks of immune evasion and dysfunction, and a reduced response to immune checkpoint inhibitors (ICIs) (61). Another study also provides evidence that GC patients with elevated lactylation levels tend to have a worse prognosis (51). Mechanistic studies indicate that lactylation modification at the K90 site on Yes-associated protein (YAP) and the K108 site on TEA domain transcription factor 1 (TEAD1) enhances the nuclear localization and stability of the YAP-TEAD transcriptional complex. This activation of the Hippo signaling pathway ultimately stimulates gastric cancer cell growth (12). Moreover, H3K18la promotes the transcription of vascular cell adhesion molecule 1 (*VCAM1*), thereby mediating the activation of the AKT-mTOR signaling pathway. This in turn facilitates the recruitment of mesenchymal stem cells mediated by C-X-C motif chemokine ligand 1 (CXCL1), remodels the TME, and accelerates tumor progression (62). These studies demonstrate that lactylation is closely linked to GC progression, suggesting that targeting lactylation may be a promising strategy for cancer therapy, including ICI treatment.

#### 4.1.2 Hepatocellular carcinoma

Hepatocellular carcinoma (HCC) ranks as the sixth most common cancer globally and is the fourth leading cause of cancer-related deaths, with a five-year survival rate of only 12.5% (63, 64). Research has revealed that L-lactate and D-lactate regulate HCC through distinct mechanisms. D-lactate, produced by intestinal microbes, functions as an endogenous immunomodulator, encouraging the shift of M2-type TAMs toward the M1 phenotype, thereby reshaping the immunosuppressive tumor microenvironment of HCC (65). On the other hand, L-lactate mediates lactylation modifications at the H3K56 site of histones and the K230 and K322 sites of non-histone ALDOA. This leads to the dissociation of ALDOA from DEAD-box helicase 17 (DDX17), enabling DDX17 to enter the nucleus and upregulate the expression of the transcription factor SOX2. This, in turn, enhances the proliferation, migration, glycolysis, and tumorigenicity of liver cancer stem cells (LCSCs) (66). Additional research has highlighted the involvement of lactylation in the development of liver cancer. Lactylation of histone H3K9 and H3K56 accelerates tumor progression by upregulating cell cycle-related genes (43). Lactylation at the K348 site of cyclin E2 (CCNE2) serves as a critical regulatory node, promoting the growth of tumor cells. SIRT3 can remove the lactylation modification of CCNE2, thereby inducing apoptosis in hepatocellular carcinoma cells (67). In addition, lactylation at the K28 site of the metabolic enzyme adenylate kinase 2 (AK2) inhibits its enzymatic activity, thereby enhancing the growth and spread of hepatocellular carcinoma (49).

#### 4.1.3 Pancreatic ductal adenocarcinoma

Pancreatic ductal adenocarcinoma, originating from the ductal epithelium of the pancreas, is characterized by its highly aggressive nature and consequently elevated mortality rate, earning it the reputation as the “king of cancers” (68–70). The 2020 global cancer statistics show that the number of new pancreatic cancer cases is nearly equal to the number of deaths, with around 490,000 cases reported (71). The occurrence of pancreatic cancer is associated with various factors such as chronic inflammation, pancreatic cysts, diabetes, and genetic predisposition (72, 73). From a molecular perspective, alterations in energy metabolism are closely linked to pancreatic pathology, since the growth of pancreatic tumor cells is highly dependent on both oxidative phosphorylation and glycolysis (74, 75). Recent studies have linked lactylation modification to the onset and development of pancreatic cancer.

The H3K18la modification in the promoter regions of TTK protein kinase (*TTK)* and BUB1 mitotic checkpoint serine/threonine kinase B (*BUB1B*) promotes their transcription and enhances glycolysis. This process further enhances the phosphorylation of P300 and tyrosine 239 (Y239) of LDHA, thus creating a reinforcing cycle involving glycolysis, H3K18la, and TTK/BUB1B, which drives the malignant progression of pancreatic cancer (76). Moreover, lactylation at the K91 site of transcription factor EB (TFEB) prevents its interaction with WW domain-containing E3 ubiquitin protein ligase 2 (WWP2), thereby inhibiting TFEB ubiquitination and subsequent degradation by the proteasome. This process enhances TFEB function and promotes autophagic flow (77). Nucleolar and spindle associated protein 1 (NUSAP1) promotes lactate accumulation through the glycolysis pathway mediated by LDHA, which in turn positively feedbacks to enhance the lactylation modification of NUSAP1 itself, thereby stabilizing the NUSAP1 protein and exacerbating the malignant phenotype of tumors (78). Furthermore, lactylation modification at the K128 site of the nicotinamide mucleotide adenylyltransferase 1 (NMNAT1) mediated by P300 not only promotes its nuclear localization and enzymatic functions but also ensures the survival of tumor cells in glucose-deprived conditions by maintaining the nuclear NAD^+^ salvage pathway (79).

#### 4.1.4 Colorectal cancer

Colorectal cancer is among the three most common malignancies worldwide in terms of incidence rates, and its mortality rate ranks second among all tumors (71). Metabolic reprogramming is closely associated with the onset and progression of colorectal cancer. Recent studies have shown that O-GlcNAc modification at the S415 site increases the stability of the c-Myc protein, which in turn activates PDK2 expression, disrupts the TCA cycle, reduces ROS production, and accelerates the proliferation of colorectal cancer cells, thereby supporting tumor growth (80). SP1-induced HIF1A-AS2 can facilitate the aerobic glycolysis and advancement of colorectal cancer through the miR-141-3p/FOXC1 axis (81). Lactylation, acting as a bridge between metabolic reprogramming and epigenetics, has also been identified as a key factor in the initiation and advancement of colorectal cancer.

The GPR37 activates the Hippo pathway, which in turn upregulates the expression of LDHA and enhances glycolytic activity. This ultimately increases the transcription of *CXCL1* and *CXCL5* by elevating the level of H3K18la modification, thereby driving liver metastasis of colorectal cancer (82). The H3K18la modification enhances transcription of rubicon-like autophagy enhancer (*RUBCNL/Pacer*), mediating the recruitment of the PtdIns3K complex through interaction with Beclin 1 (BECN1), thereby promoting the maturation of autophagosomes and enhancing tumor cell resistance to bevacizumab (83). Furthermore, the attenuation of expression of structural maintenance of chromosomes protein 4 (SMC4) promotes the expression of hexokinase 2 (*HK2*), phosphofructokinase (*PFKL*), and *ALDOC*, while inhibiting the expression of phosphoglycerate mutase 1 (PGAM1), leading to lactate accumulation. This process elevates the levels of ATP-binding cassette transporter through H4K12la, thereby diminishing the chemosensitivity of cancer cells (84). The lactylation of meiotic recombination 11 homolog A (MRE11) at the K673 site enhances its binding to DNA, facilitating DNA end resection and homologous recombination (HR). This modification promotes chemoresistance in cancer cells, including colorectal cancer, by improving DNA damage repair (85). At the non-histone level, lactylation at the K408 site of the eEF1A2 promotes tumor progression by enhancing protein synthesis activity (33). Proprotein convertase subtilisin/kexin 9 (PCSK9) mediates epithelial-mesenchymal transition (EMT) in colon tumor cells by upregulating snail homolog 1 (Snail 1), downregulating E-cadherin expression, and simultaneously upregulating N-cadherin and matrix metallopeptidase 9 (MMP9) expression, thereby promoting tumor metastasis (86). The H3K18la modification in macrophages inhibits the expression of retinoic acid receptor γ (*RARγ*), activating the TRAF6-IL-6-STAT3 signaling pathway, thus promoting the onset and progression of colorectal cancer (87).

#### 4.1.5 Esophageal cancer and cholangiocarcinoma

Lactylation also plays a role in the progression and advancement of esophageal cancer and cholangiocarcinoma. In esophageal cancer, hypoxia-induced enhancement of histone H3K9la specifically increases the transcriptional activity of laminin subunit gamma 2 (*LAMC2*), thereby promoting tumor proliferation and invasion (88). In cholangiocarcinoma, lactylation at the K477 site of nucleolin (NCL) enhances MADD protein translation activity by modulating MADD RNA splicing. This, in turn, activates the MEK/ERK signaling pathway and accelerates tumor progression (89).

### 4.2 Regulatory network of lactylation in urogenital system tumors

**Figure 3:**
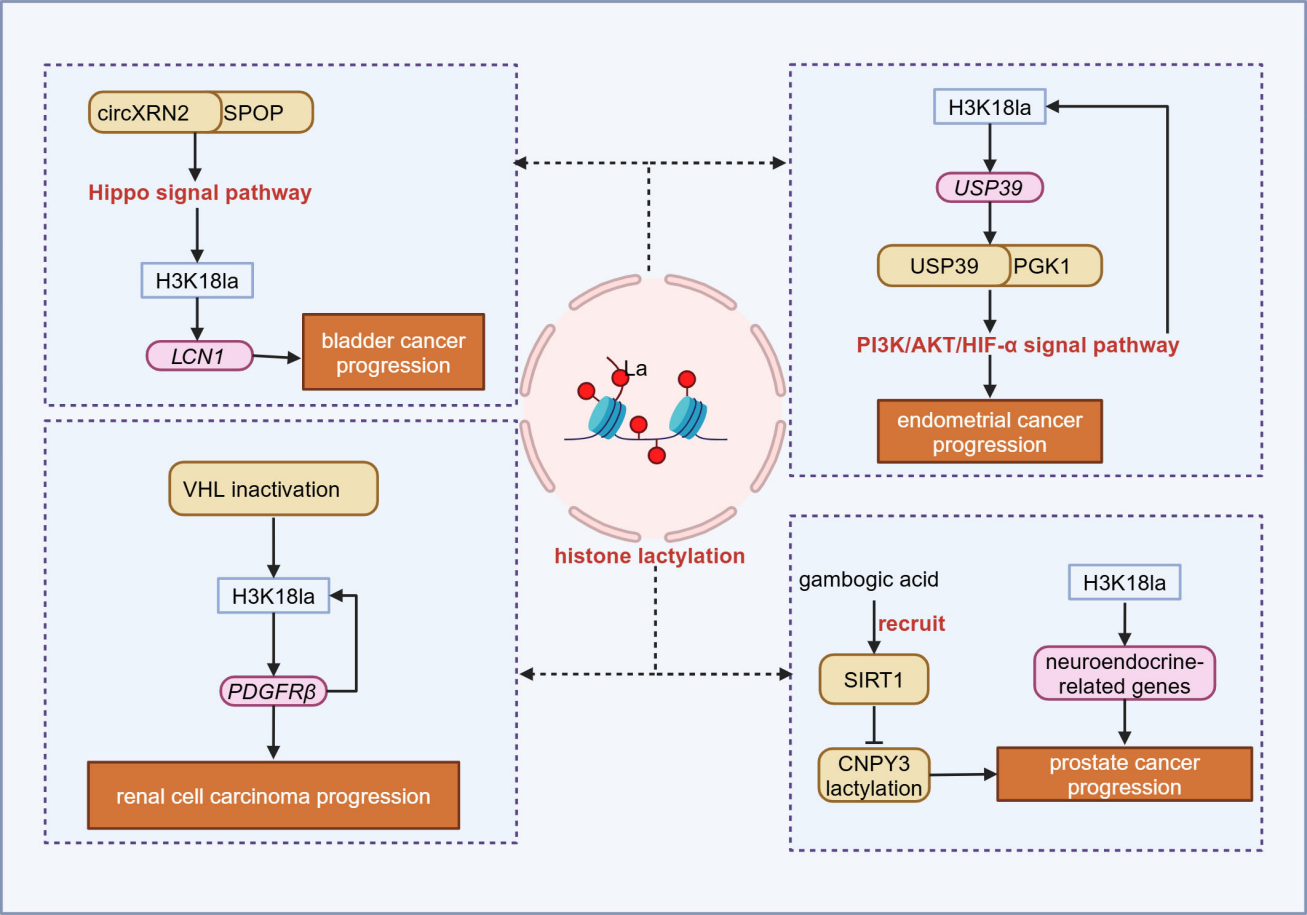
Lactylation in urogenital system tumor. In the urogenital system, histone lactylation can promote tumor progression by inducing gene transcription and activating downstream signaling. SPOP, Speckle-type POZ; LCN1, Lipocalin 1; VHL, Von hippel-lindau; PDGFRβ, Platelet-derived growth factor receptor β; USP39, Ubiquitin specific peptidase 39; PGK1, Phosphoglycerate kinase 1; SIRT1, Sirtuin 1; CNPY3, Canopy FGF signaling regulator 3. Figure created in https://BioRender.com.

In bladder cancer, circular RNA circXRN2 interacts with speckle-type POZ (SPOP) to activate the Hippo signaling pathway, inhibiting H3K18la of the lipocalin-2 (*LCN2*) gene promoter region, thereby downregulating *LCN2* expression and suppressing tumor progression (90). In renal cell carcinoma, the inactivation of Von Hippel-Lindau (VHL) promotes the H3K18la modification of platelet-derived growth factor receptor β (*PDGFRβ*), activating its transcription and driving tumor progression by H3K18la-PDGFRβ positive feedback loop (91). In prostate cancer, lactate accumulation promotes the transcription of neuroendocrine-related genes through H3K18la modification, inducing neuroendocrine differentiation of adenocarcinoma (92). Moreover, gambogic acid can inhibit the lactylation of canopy FGF signaling regulator 3 (CNPY3) by recruiting SIRT1, thereby inducing lysosomal rupture and initiating pyroptosis in tumor cells (93). Research on endometrial cancer reveals that H3K18la modification in the promoter region of ubiquitin specific peptidase 39 (*USP39*) upregulates its expression. USP39 interacts with PGK1, thereby activating the PI3K/AKT/HIF-1α signaling pathway, enhancing glycolysis and lactylation, and establishing a carcinogenic positive feedback cycle (94) (**Figure 3**).

### 4.3 Regulatory network of lactylation in lung cancer

In NSCLC, H3K18la levels are higher in tumor tissues than in adjacent tissues and are positively correlated with the clinical and primary tumor (T) stages of patients. This suggests that patients with higher lactylation levels have a worse prognosis (95). At the mechanistic level, histone lactylation promotes the expression of the glycolysis-related gene hexokinase 1 (*HK-1*) while inhibiting the expression of the gene isocitrate dehydrogenase three non-catalytic subunit γ (*IDH3G*), maintaining mitochondrial homeostasis and driving tumor progression (40). Furthermore, inactivation of the Numb/Parkin pathway impairs mitophagy and leads to metabolic reprogramming. This process promotes the transcription of neuroendocrine-related genes by increasing H3K18la levels, ultimately resulting in the transdifferentiation of lung adenocarcinoma into neuroendocrine tumors (92). These results indicate that lactate can regulate cellular metabolism through histone lactylation, thereby promoting the malignant progression of tumors. H3K18la can also promote the transcription of absent in melanoma 2 (*AIM2*), thereby inhibiting the phosphorylation of STAT5B and promoting its proteasomal degradation, ultimately weakening the transcriptional promotion effect of STAT5B on Acyl-CoA synthetase long-chain family member 4 (*ACSL4*), thereby facilitating the progression of lung cancer (96). Additionally, telomerase reverse transcriptase (TERT), as one of the key catalytic subunits of telomerase, can prevent replicative senescence damage caused by DNA shortening by extending telomeres. The tumor suppressor LKB1 can inhibit the transcriptional activity of specificity protein 1 (SP1) by downregulating H4K8la and H4K16la, thereby inhibiting the transcriptional expression of TERT and inducing senescence in lung adenocarcinoma cells (97). Aldo-keto reductase family 1 member B10 (AKR1B10) enhances glycolysis through regulation of LDHA expression and boosts Cyclin B1 (CCNB1) transcription by elevating H4K12la levels. These actions speed up DNA replication and cell cycle progression, while also enhancing pemetrexed resistance in lung cancer cells with brain metastasis (98). Overall, histone lactylation can influence NSCLC energy metabolism, cellular senescence, and chemotherapy resistance by regulating gene expression, thereby promoting tumor progression.

Apart from the aforementioned mechanisms, non-histone lactylation can also promote lung cancer progression. BZW2 promotes lactate production through glycolysis and facilitates the lactylation of IDH3G, which in turn drives the progression of lung adenocarcinoma (99). Lactylation of sex determining region Y box protein 9 (SOX9) can enhance the glycolysis process, thereby promoting the stemness, migration, and invasion of NSCLC cells (100). Furthermore, lactate-induced lactylation of insulin-like growth factor 1 receptor (IGF1R) increases its protein stability and promotes its binding to IGF1, which accelerates lung cancer proliferation (101). These results indicate that non-histone lactylation drives the malignant progression of lung cancer by boosting glycolytic activity and stabilizing proteins.

**Figure 4:**
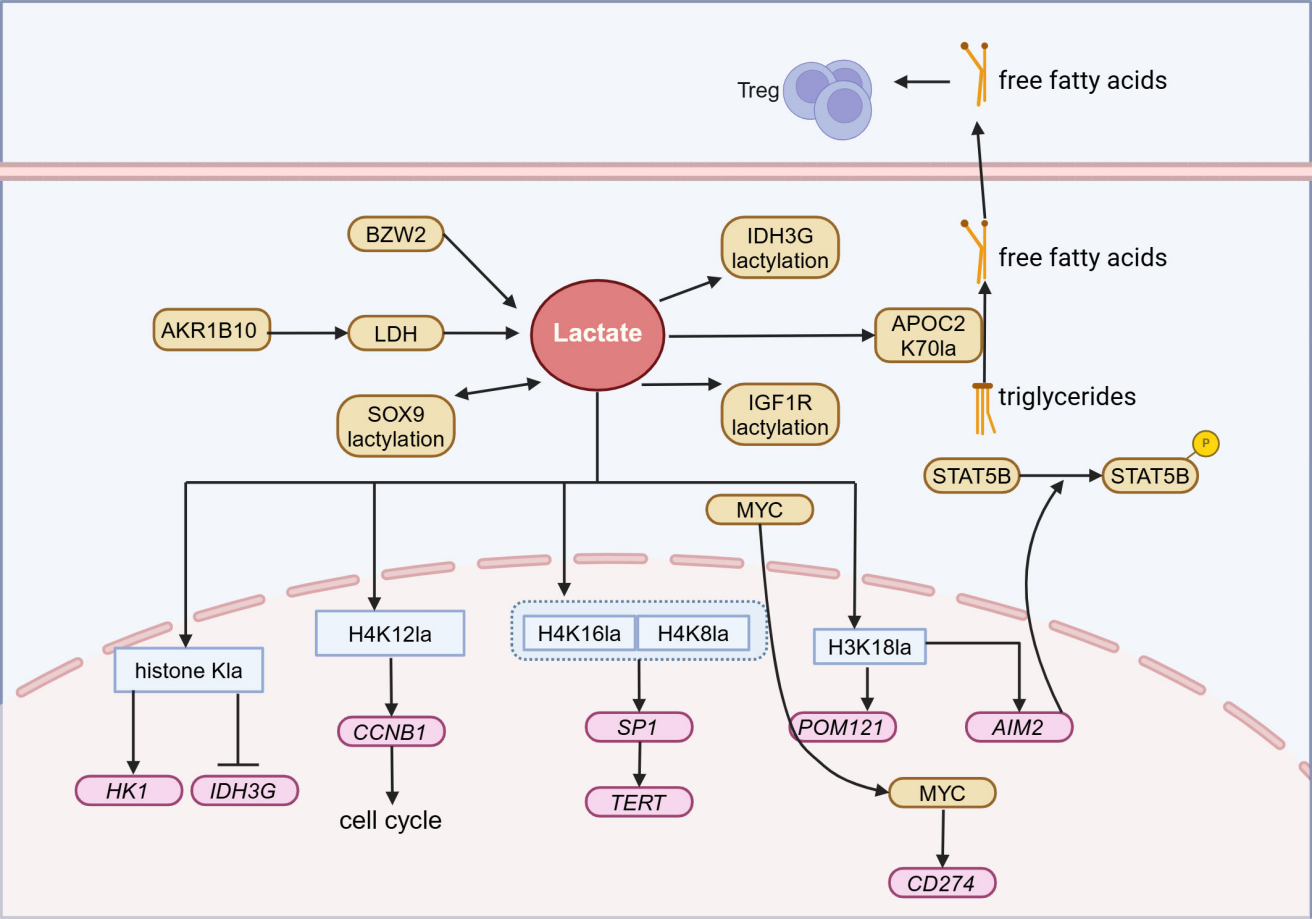
Lactylation in Lung cancer. In lung cancer, lactate can participate in the energy metabolism, chemoresistance, and immune evasion of lung cancer through histone and non-histone lactylation modifications, ultimately leading to tumor progression. Treg, Regulatory T cells; AKR1B10, Aldo-keto reductase family 1 B10; BZW2, Basic leucine zipper and W2 domains 2; LDH, Lactate dehydrogenase; SOX9, Sex determining region Y box protein 9; IDH3G, Isocitrate dehydrogenase three non – catalytic subunit gamma; APOC2, Apolipoprotein C2; IGF1R, Insulin-like growth factor 1 receptor; MYC, MYC proto-oncogene; HK1, Hexokinase 1; CCNB1, Cyclin B1; SP1, Specificity protein 1; TERT, Telomerase reverse transcriptase; POM121, Pore membrane protein 121; AIM2, Absent in melanoma 2. Figure created in https://BioRender.com.

Epigenetic changes, particularly the novel protein post-translational modification known as lactylation, have been revealed as one of the key mechanisms of resistance to immunotherapy in NSCLC (102). In NSCLC, H3K18la not only promotes immune evasion by inhibiting the cytotoxicity of CTLs but also directly activates the transcription of pore membrane protein 121 (*POM121*), enhances the nuclear transport of MYC, and induces programmed cell death ligand 1 (PD-L1) expression, thereby exacerbating immune resistance (95). Moreover, lactylation at the K70 site can stabilize apolipoprotein C2 (APOC2), facilitating the conversion of triglycerides into free fatty acids and their release extracellularly, thereby driving the proliferation of regulatory T cells (Tregs) and enhancing tumor resilience against anti-PD-1 therapy (103) (**Figure 4**).

### 4.4 Regulatory network of lactylation in other tumors

#### 4.4.1 Uveal melanoma

The H3K18la modification promotes the transcription of *YTHDF2*, accelerating the mRNA degradation of m6A-modified period circadian regulator 1 (PER1) and tumor protein p53 (TP53), thereby promoting tumorigenesis (32). Another mechanism suggests that histone lactylation can remove N1-methyladenosine (m1A) methylation of the speckled protein 100A (SP100A), upregulate AlkB homolog 3 (ALKBH3) expression, and inhibit the formation of promyelocytic leukemia (PML) aggregates, thereby synergistically driving tumor progression (104).

#### 4.4.2 Glioma

In glioma, elevated lactate levels induce the lactylation modification of CD73 in tumor cells, CD39 and chemokine receptor CCR8 in Tregs, CD39 in macrophage, and CD73 in T cells, reshaping the pro-tumor immune phenotype (105). In an important type of glioma—glioblastoma, the H3K18la modification upregulates the long non-coding RNA LINC01127, guiding POLR2A to the promoter region of MAP4K4, activating the JNK pathway and promoting the self-renewal of tumor stem cells (106). Overexpression of aldehyde dehydrogenase 1 family member A3 (ALDH1A3) promotes PKM2 tetramerization and enhances the lactylation of X-ray repair cross-complementing protein 1 (XRCC1) at the K247 site. Lactylation modification induces the neutralization of surface charge on XRCC1, thereby increasing the nuclear translocation of XRCC1 by promoting its binding with IMPORTINα, leading to radioresistance through enhanced DNA repair capability (107). Moreover, histone lactylation modification in macrophages within the TME can induce T cell exhaustion by upregulating IL-10 expression, which synergistically promotes tumor progression (108).

#### 4.4.3 Breast cancer

The lactate accumulation upregulates the transcriptional expression of serine/arginine splicing factor 10 (SRSF10) by modifying the promoter region of the oncogene c-Myc with H3K18la. This process drives the alternative splicing of murine double minute 4 (MDM4) and B-cell leukemia-X protein (Bcl-x) in breast cancer cells, thereby facilitating tumor progression (109). Another study shows that potassium channel, subfamily K, member 1 gene (KCNK1) enhances glycolysis in breast cancer cells by interacting with and activating LDHA, which subsequently induces the expression of ZW10 interactor (*ZWINT*), *Anillin*, actin binding protein (*ANLN*), and *LDHA* through H3K18la modification, promoting proliferation, invasion, and metastasis (110).

#### 4.4.4 Thyroid cancer and hematologic tumors

In studies of undifferentiated thyroid cancer, the BRAFV600E mutation can enhance glycolysis and H4K12la modification, upregulating proliferation-associated genes, including connective tissue growth factor (*CTGF*), cyclin-dependent kinase 1 (*CDK1*), cyclin E1 (*CCNE1*), kruppel-like factor 2 (*KLF2*), *IL1β*, and aurora kinase B (AURKB) (111). In acute myeloid leukemia (AML), a high level of STAT5 expression drives histone lactylation, upregulates the transcription of PD-L1, and induces CD8+ T cell exhaustion, indicating that modulating the PD-1/PD-L1 axis might counteract STAT5-induced immunosuppression (112).

### 4.5 Lactylation modification reshapes the TME

The TME is a constantly evolving and diverse environment that supports tumor growth and expansion (9). Lactylation can drive tumor progression by modifying the functions and signaling pathways of immune cells.

Lactate upregulates the expression of methyltransferase like 3 (METTL3) in TIM via H3K18la modification. Lactylation at the K281 and K345 sites of METTL3 enhances its RNA-binding capacity, thereby promoting the immunosuppressive function of TIM through the METTL3-JAK1-STAT3 signaling axis (21). Moreover, a high lactate environment can promote lactylation at the K72 site of MOESIN in Treg cells, thereby enhancing TGF-β signaling transduction to maintain an immunosuppressive phenotype (113). Lactylation modification has been found to regulate cytokine release or immune cell function, thereby influencing anti-tumor immune effect. In macrophages, lactate increases the cytokine concentrations including IL-6 and IL-10 in the TME by enhancing the level of H3K18la. This drives macrophages toward an immunosuppressive phenotype and leads to T cells being in an immunosuppressive state, ultimately inhibiting the anti-tumor effect (87, 108).

In summary, lactylation modifications drive tumor progression through multiple mechanisms, on one hand by modulating the activity of tumor-related genes and proteins, and on the other hand by reshaping the immunosuppressive microenvironment. Analyzing the spatiotemporal specificity of lactylation modifications will provide new strategies for targeted interventions.

## 5 Lactylation as a diagnostic and prognostic indicator in tumors

Lactylation not only characterizes the abnormal metabolism of cells but also demonstrates unique clinical and prognostic value by directly participating in pathological processes. Multiple studies have confirmed that lactylation levels correlate strongly with clinical analyses, therapeutic responses, and survival outcomes in various tumors.

Studies have shown that H3K18la can serve as a diagnostic and prognostic biomarker for epithelial ovarian cancer and pancreatic cancer, while H4K12la can act as a prognostic biomarker for triple-negative breast cancer (114–116). The overall lactylation level can be used as a prognostic biomarker for gastric cancer (**Table 5**) (51). In addition, lactylation-related gene models constructed based on multi-omics data have demonstrated diagnostic and prognostic prediction capabilities in renal cell carcinoma, skin melanoma, gastric cancer, breast cancer, pancreatic cancer, multiple myeloma, hepatocellular carcinoma, ovarian cancer, nasopharyngeal cancer, and prostate cancer (117–131). However, their clinical translation still requires addressing key issues such as the limitations of single-center studies, the standardization of detection technologies, and the dynamic regulation mechanisms of the metabolic microenvironment.

**Table 5:** Lactylation as a tumor diagnostic and prognostic indicator.

Tumor type	The clinical relevance of lactylation	Diagnostic or prognostic indicator	Reference
Epithelial ovarian cancer	High H3K18la level is strongly correlated to advanced tumor staging (*P* = 0.037), early relapse after platinum-based therapy (*P* = 0.002), and shortened OS (*P* = 0.028) and PFS (*P* < 0.001)	Prognostic indicator	(115)
Pancreatic cancer	High H3K18la level is significantly positively correlated with CA19-9 (*P* < 0.001) and CEA (*P* < 0.01)	Diagnostic indicator	(116)
Triple-negative breast cancer	High H4K12la level is significantly associated with an increased Ki-67 proliferation index (*P* = 0.0027) and shortened OS (*P* = 0.0164)	Prognostic indicator	(114)
Gastric cancer	High lactylation level is linked to higher clinical staging, lower differentiation, a greater propensity for lymph node metastasis, and shorter OS (*P* < 0.001)	Prognostic indicator	(51)

## 6 Therapeutic strategies targeting lactylation in tumor treatment

Therapeutic strategies targeting lactylation modifications focus on regulating lactate metabolic nodes and modification enzyme systems.

### 6.1 Regulation of lactylation modifications by targeting lactate production and transport processes

In terms of targeting lactate metabolic nodes, inhibiting key enzymes like LDH and the transport system MCT can significantly impact disease progression.

Currently, several anti-LDH inhibitors are under development and are being evaluated in preclinical studies. Studies have shown that combining anti-PD-1 treatment with the LDHA inhibitor GSK2837808A demonstrates more potent anti-tumor efficacy than using anti-PD-1 antibody alone. Mechanistic studies reveal that the reduction of lactate inhibits the number of Tregs and tumor growth, thereby enhancing anti-tumor immune response (113). Another LDH inhibitor, GNE-140, has also been proven to inhibit the proliferation of mouse melanoma and human pancreatic cancer cells *in vitro* (132). Additionally, chemicals and natural products, including oxalate, crocetin, gossypol, vitamin C, and royal jelly acid, have also been reported to slow disease progression by inhibiting LDH (132, 133).

Various MCT inhibitors, such as syrosingopine, AR-C155858, 7ACC2, BAY8002, SR13800, and AZD3965, have been demonstrated to suppress tumors like multiple myeloma, pancreatic cancer, and Parkinson’s disease by inhibiting MCT activity (134–136). Among them, the MCT1/MCT2 inhibitor AZD3965 is currently in phase I clinical trials (NCT01791595) targeting late-stage solid tumors and diffuse large B-cell lymphoma. Preclinical studies have confirmed its efficacy against lung cancer, bladder cancer, and lymphoma (137–139).

Moreover, GTP-specific SCS (GTPSCS) and acetyl-CoA synthetase 2 (ACSS2), which function as Lactyl-CoA synthetases, are poised to serve as emerging therapeutic targets for lactylation-associated tumors (11, 140). However, their distribution and specific mechanisms of action remain unclear and require further exploration to potentially aid in disease treatment.

### 6.2 Targeting lactylation regulatory enzymes

In terms of targeting modification enzymes, drugs targeting lactylation modification enzymes and demodification enzymes have shown therapeutic potential for various diseases. In the study of undifferentiated thyroid cancer, the p300 inhibitor C646 and the Raf inhibitor PLX4032 exhibit synergistic effects, with their combined antitumor activity being superior to that of a single drug (111). The GCN5 inhibitor CPTH6 suppresses the growth of lung cancer stem-like cells and reduces the viability of both these cells and other cancer cell lines (141). Moreover, targeting demodification enzymes can also treat various diseases. For example, brief treatment with the HDAC inhibitor trichostatin A can effectively inhibit nasopharyngeal carcinoma cell proliferation and induce epithelial-mesenchymal transition, without enhancing their invasive ability (142). In hepatocellular carcinoma, the SIRT3 activator Honokiol has been shown to exert antitumor effects by directly activating lactylation demodification enzymes (67). In studies on gastric cancer, the SIRT2 inhibitor AGK2, when used in conjunction with the copper ion carrier Elesclomol, can promote copper-induced cancer cell death (143). These studies indicate the vast potential of targeting enzymes regulating lactylation in tumor treatment.

It is crucial to note that p300, HDACs, and SIRTs, as core enzymes in epigenetic regulation, participate in multiple epigenetic modifications beyond lactylation, including acetylation (10, 144). Therefore, when developing specific drugs targeting lactylation modification, it is crucial to strictly avoid interference with non-target modification pathways like acetylation, thereby reducing off-target effects and potential toxicity. To address this, structural biology methods can be employed to analyze the three-dimensional spatial conformation of p300/HDACs/SIRTs and lactylated substrate complexes. Based on the topological features of their binding pockets, specific small-molecule inhibitors or allosteric modulators can be developed. For instance, AlphaFold can be utilized to predict dynamic conformational changes in the lactylation binding domain, optimizing the spatial compatibility between drug molecules and targets to enhance selectivity (145). Additionally, systematic identification of specific regulatory networks for lactylation modification can be achieved through CRISPR-Cas9 whole-genome screening technology (146). By excluding cross-regulatory pathways with acetylation, disease-related targets can be further identified, and non-specific effects can be avoided.

Furthermore, lactylation not only drives tumorigenesis and progression but also participates in physiological processes, such as the regulation of inflammatory responses, maintenance of embryonic stem cell pluripotency, and osteoblast differentiation. This suggests that non-selective inhibition of lactylation may lead to severe off-target toxicity due to interference with normal physiological functions. To achieve therapeutic specificity, it is necessary to prioritize the screening of abnormally activated lactylation targets in pathological states. For instance, CircXRN2 can specifically downregulate the expression of *LCN2* in bladder cancer cells by inhibiting H3K18la, thereby inhibiting tumor progression. Additionally, the development of proteolysis-targeting chimeras (PROTACs) enables the selective degradation of pathogenic lactylated proteins through the ubiquitin-proteasome system, circumventing the developmental or metabolic toxicity caused by global inhibition (147). This approach may offer a new direction for balancing efficacy and safety.

Overall, lactylation is gaining widespread attention as a new target for tumor therapy. By targeting lactylation modification, it is possible to regulate tumor progression and restore immune function, providing new strategies for tumor treatment. With further research, the clinical application of drugs targeting lactylation is expected to offer more effective treatment options for patients and aid in precision therapy.

## 7 Conclusion

As a nexus linking metabolic reprogramming and epigenetic regulation of tumors, the study of the molecular mechanisms of lactylation opens a new paradigm for tumor diagnosis and treatment. Lactylation modification is deeply involved in tumor pathology by dynamically modulating gene expression and protein activity. Prognostic models based on lactylation modification levels have shown clinical value in tumors. Additionally, intervention strategies that target metabolic nodes and modification enzymes offer new insights for precision medicine. However, critical issues such as the substrate recognition mechanism of lactylation modification enzymes, the functional heterogeneity of isomers, and the spatiotemporal regulation of metabolic flux in the microenvironment urgently need breakthroughs. By integrating single-cell multi-omics and organoid models, we systematically analyze the dynamic network of lactylation modifications, laying the theoretical foundation for developing metabolism-epigenetics combined therapeutic strategies, and ultimately promoting the transition from exploratory research to clinical translation.
